# Incubators for Infants

**Published:** 1887-03-05

**Authors:** R. A. Owthwaite

**Affiliations:** Secretary. City of London Lying-in-Hospital, City Road, E.C.


### INCUBATORS FOR INFANTS.

I notice, on page 371 of this week's Hospital, you draw attention to the Incubator in use at the Glasgow Maternity Hospital. Perhaps you are not aware that an incubator on a much larger scale has been in use in this Hospital for over three years, and has been found of the greatest service in maintaining animation in premature or apparently still-born children; in addition to this, its advantages cannot be too highly spoken of as a means of quieting cross babies. The matron will be pleased to show the Incubator, and explain its working, to any medical man or matron any morning before 12 o'clock.

R. A. Owthwaite, Secretary.
City of London Lying-in-Hospital, City Road, E.C.

